# Thrombin as a Potential Proxy to Select for Horn Fly Abundance in Beef Cattle

**DOI:** 10.3390/ani12212982

**Published:** 2022-10-30

**Authors:** Amanda Warner, Ashley Ling, Taylor Krause, Bradley Heins, Nancy Hinkle, Dean Pringle, Samuel E. Aggrey, Romdhane Rekaya

**Affiliations:** 1 Department of Animal and Dairy Science, University of Georgia, Athens, GA 30602, USA; 2 College of Veterinary Medicine, University of Georgia, Athens, GA 30602, USA; 3 Department of Entomology, University of Georgia, Athens, GA 30602, USA; 4 Department of Poultry Science, University of Georgia, Athens, GA 30602, USA

**Keywords:** horn flies, thrombin, heritability, genetic selection

## Abstract

**Simple Summary:**

Horn flies are a major nuisance to livestock, resulting in reduced productivity and substantial economic losses. Current fly control methods have temporary efficacy, adversely impact the environment, and increase fly resistance to insecticides. Using the animal’s innate resistance and tolerance to horn flies through genetic selection could be an attractive alternative. Unfortunately, measuring horn fly abundance, especially under pasture conditions, is economically and logistically challenging, and alternative approaches are needed. In this study, thrombin, a major player in blood coagulation, was investigated as a potential proxy trait to assess on-animal fly counts. Our genetic analyses showed that the blood thrombin level is negatively correlated with fly count, is moderately heritable, and can be used to select against fly abundance in beef cattle.

**Abstract:**

Horn flies are a major nuisance to cattle and induce significant economic losses. Fly abundance varies within and across breeds and genetic analyses have shown sufficient genetic variation to permit selection. A major bottleneck for selecting against horn fly abundance is the complexity of measuring fly attraction phenotypes. Easy-to-measure proxy phenotypes could be an attractive option to indirectly estimate fly abundance. In the current study, thrombin was investigated as a potential proxy to assess fly abundance. Fly counts and blood samples were collected on 355 cows. Pearson correlation between subjective fly count and thrombin was −0.13, indicating a decrease in fly abundance with the increase in thrombin concentration. When thrombin was discretized into three classes, there was a 22% difference in fly count between the top and bottom classes. Heritability estimates of thrombin were 0.38 and 0.39 using linear and threshold models, respectively. The correlation between estimated thrombin breeding values and fly count was around −0.18. There was a noticeably lower density of high fly counts among animals with high breeding values for thrombin. These results indicate that thrombin could be used in combination with other biological factors to estimate fly abundance and as a proxy for selection against fly abundance.

## 1. Introduction

Horn flies, *Haematobia irritans*, are one of the most prevalent pests on pasture-dwelling cattle [1]. Horn flies (HF) are a major irritant to cattle as they are obligate blood feeders and can consume 20 to 38 blood meals per day [2]. While the average HF blood meal size is only 1.5 mg, there can be upwards of 1000 horn flies feeding on an animal at a given time [3]. HF blood feeding can cause stress, increased heart and respiratory rates, decreased weight gain and feed efficiency, decreased weaning weights, and reduced milk production in cattle [4,5,6,7,8]. Additionally, fly avoidance behaviors in cattle, such as tail flicks, leg stomps, skin twitches, head throws, and increased movement during grazing, could lead to further reductions in feed efficiency [9]. Collectively, these factors make horn flies one of the most economically detrimental pests on pastured cattle. In the United States, horn flies have been estimated to cause more than $1 billion dollars annually in economic losses [10]. Disturbing the non-symbiotic relationship between HF and cattle is of critical economic, health, and animal welfare importance to the beef cattle industry.

Several control methods have been proposed to deal with HF infestation, including life cycle interruption through manure management, inclusion of insect growth regulators in the host diet, and reduction in the adult fly population by using insecticides. Although these insecticides convey a certain level of fly control, the outcome is temporary, and their efficacy is hampered by the need for multiple applications during a fly season. Insecticide control also creates economic and logistic costs, migration of flies from neighboring herds, and adverse environmental impacts. Furthermore, intensive use of insecticides has led to HF resistance to these products and a reduction in their predation by other insects [11,12,13].

Several studies [14,15] have clearly shown differences in the abundance or attraction of HF across breeds of cattle and among animals within the same breed. Genetic analyses of HF abundance traits in cattle have shown sufficient genetic variation to allow for improvement in resistance through selection, with heritability estimates ranging from 10 to 80% [16,17,18]. Similar results, though with lower heritabilities, were observed for self-reported wheal size, itch intensity, and attractiveness to mosquitoes in humans [19].

One of the major problems facing potential selection for HF resistance and tolerance is the economic and logistic costs associated with measuring fly abundance or attraction phenotypes, especially under pasture conditions. In pastures, animals are often clustered into groups with constant mobility. Some animals are not comfortable with the close proximity of humans or vehicles, which further complicates the collection of the HF data. Counts of HF load per animal by trained evaluators or digital images have been frequently used as a proxy to measure resistance and tolerance. Unfortunately, the accuracy of this approach depends largely on the quality of the subjective assessments of evaluating agents or the acquisition and processing of images. Thus, alternative approaches to assess fly abundance to better understand resistance and tolerance need to be developed. These approaches should consider non-count-based phenotypes that can be cheaply, efficiently, and precisely measured.

It has been reported that differences in HF tolerance in beef cattle could be associated with a variation in blood enzymes, primarily those associated with blood clotting [20]. Thrombin (TH), a major enzyme for hemostasis that plays an important role in the activation of several pro-coagulation factors through the conversion of fibrinogen to fibrin, has been suggested to be associated with HF count in cattle [21,22]. Although the assumption is reasonable, there is barely any meaningful field data that clearly support such a hypothesis. Studies in humans reported moderate (>0.3) to high heritability of thrombin and related phenotypes. The objectives of this study were to (1) assess the potential association between thrombin and horn fly abundance and (2) estimate the genetic parameters of thrombin to assess its adequacy as a potential proxy for the selection of HF resistance and tolerance in beef cattle.

## 2. Materials and Methods

All data used in this project were collected following the Animal Use and Care Protocol approved by the Institutional Animal Care and Use Committee at University of Georgia. Two University of Georgia farms (Eatonton Beef Research Unit and Northwest Georgia REC NWREC in Calhoun) participated in this project.

*Quantifying horn fly abundance using subjective assessment*: Subjective horn fly abundance was collected on 355 cows and heifers housed in two University of Georgia affiliated farms (NWREC and Eatonton Beef Research Unit). Most of the animals were sampled twice during June and August of 2019. Animals were not treated or managed in any way to control HF in 2019 prior to data collection. Every effort was made to minimize the disturbance of the animals on the pasture. Each animal was assessed subjectively for the abundance of horn flies by at least one of the trained technicians. Environmental conditions, including temperature, humidity, and wind speed, were recorded. Animals scored for HF abundance by more than one agent were used to assess the consistency across trained evaluators. Animals with more than one score were identified and those with differences between evaluators of less than 500 flies were kept. This was necessary to remove records where the assessment by the different evaluators was likely to have been conducted under markedly different conditions (e.g., animal moved, disturbing the flies on its back).

*Thrombin assessment:* Thrombin was assessed on 355 cows with horn fly abundance measurements. Blood samples were collected from the tail of animals evaluated for horn fly abundance roughly one week after data collection. Blood samples were processed as quickly as possible (less than one day) and serum samples were stored at −80 °C. These samples were later used to quantify thrombin in the serum using an ELISA assay (MyBioSource, San Diego, CA, USA). To determine serum thrombin content, each serum sample was measured twice. Standard samples with known thrombin concentration were measured twice and were used to establish the relationship between thrombin and the ELISA assay optical density (OD) reads. A quadratic regression on the average of OD (across the measurements for each sample) was fitted and estimated parameters (intercept, linear, and quadratic regression coefficients) were used to predict thrombin in the serum samples. Thrombin was predicted using the following equation:

$$TH_{i}={\hat{\beta }}_{0}+\left({\bar{OD}}_{i}\right){\hat{\beta }}_{1}+{\left({\bar{OD}}_{i}\right)}^{2}{\hat{\beta }}_{2}$$

where $TH_{i}$ and ${\bar{OD}}_{i}$ are the predicted serum thrombin concentration and average optical density reads for animal *i*. ${\hat{\beta }}_{0},\;\;{\hat{\beta }}_{1},\;\mathrm{and}\;{\hat{\beta }}_{2}$ are the estimations of the regression coefficients. Because the ELISA assays were run on three different days, the quadratic regression was separately fit for the data of each day. Estimates of the regression coefficients run on the three different days were obtained using their respective standard samples and are presented in Table 1.

*Genetics parameters of serum thrombin:* Due to the potential error associated with the assessment of HF abundance and thrombin and the fact that economic losses are theorized to occur when HF abundance exceeds a certain threshold, currently set at around 200 flies, the continuously distributed predicted thrombin was categorized into 3 classes (1 = TH > 500 ng/mL; 2 = 250 < TH < 500 ng/mL; 3 < 250 ng/mL). The genetic parameters for thrombin as a continuous and discrete trait were estimated using mixed linear and threshold models, respectively. The following mixed model was used:

y=Xβ+Zu+Wp+e

where y is either the vector of estimated thrombin (continuous trait) or the vector of liabilities in the case when thrombin was categorized into three classes (discrete trait). The vector β included the systematic effects of farm, pregnancy status, and age of the cow as a covariate. u and p are the vectors of additive and permanent effects, respectively, and e is the vector of random residual terms. X, Z, and W are known matrices with the appropriate dimensions, relating the phenotypes to the systematic, additive, and permanent effects, respectively. The linear and threshold models were implemented using BLUPF90 and THRGIBBS1F90 programs [23], respectively.

## 3. Results

**Figure 1:**
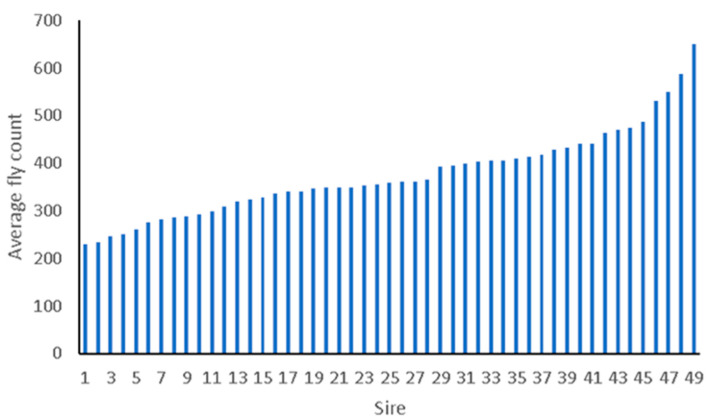
Average fly count for sire families. Only sires with 3 or more progeny were included.

Table 2 presents a summary description of the distribution of horn fly abundance at the two farms based on the subjective evaluation. For animals scored by more than one evaluator, the average was used. The cattle at Northwest Georiga REC seem to have a lower abundance, as reflected by a lower mean and a narrower spread. These are raw phenotypes, and the differences could be due to environmental factors that were assessed during the genetic analysis. On both farms, the average horn fly abundance was substantially higher than the currently accepted economic injury threshold (fly abundance at which economic loss from horn flies exceed intervention costs) of 200 flies. Furthermore, across both locations, there is a substantial variation in horn fly abundance between animals under similar management and environmental conditions (Table 2), suggesting the potential of a significant genetic component. The Pearson correlation coefficient between evaluators was 0.69. Across sire families, there has been substantial variation in horn fly abundance as indicated in Figure 1. In fact, the average fly count across sire families ranged between 230 and 650 flies.

**Figure 2:**
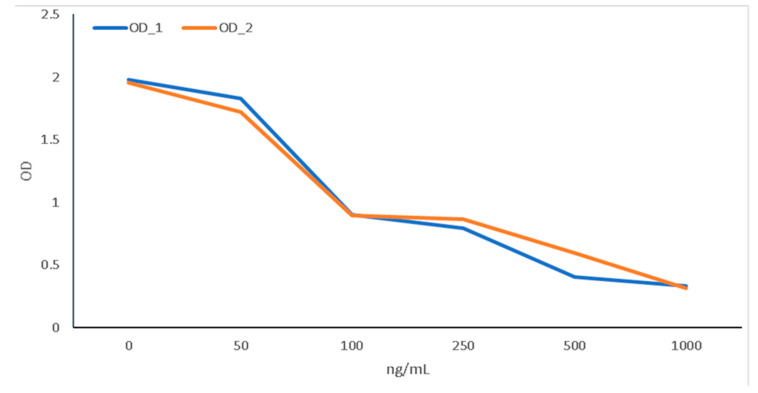
Optical density for first (OD_1) and second (OD_2) replicates of standards with known thrombin concentration (ng/mL).

Across two replicates, there was a clear consistency in the relationship between OD reads and thrombin concentration in the standards (Figure 2).

**Figure 3:**
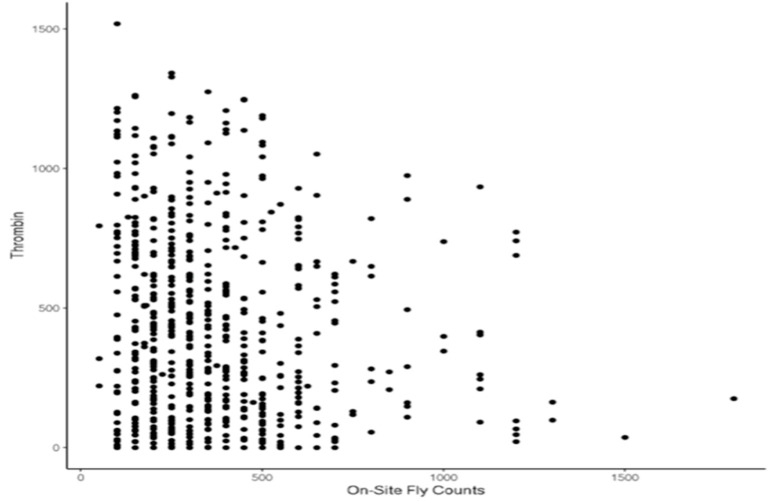
Relationship between fly counts and thrombin concentration.

The relationship was almost linear for concentrations above 100 ng/mL. However, for low thrombin concentrations (<50 ng/mL), the relationship was exponential. Figure 3 presents the distribution of predicted thrombin as a function of HF abundance. 

**Figure 4:**
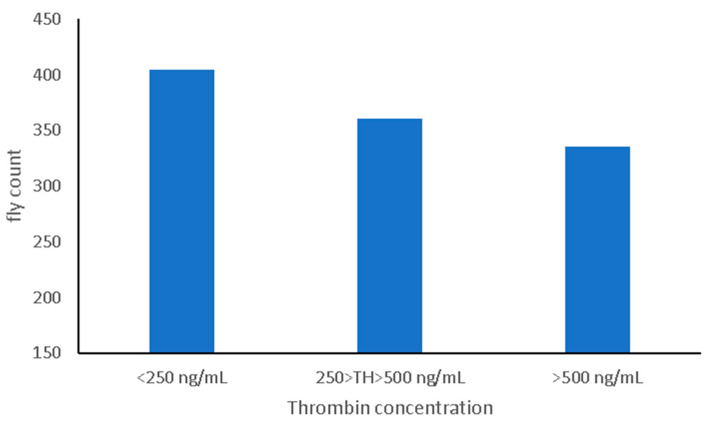
Average fly count across the three thrombin classes.

The Pearson correlation between predicted thrombin and HF count was −0.13, indicating a general decreasing trend in the number of flies with an increase in the amount of thrombin in the blood. When predicted thrombin was discretized into three classes with class boundaries chosen to achieve reasonable class size; there was a 22% difference in HF counts between the lowest and highest TH classes (Figure 4).

**Figure 5:**
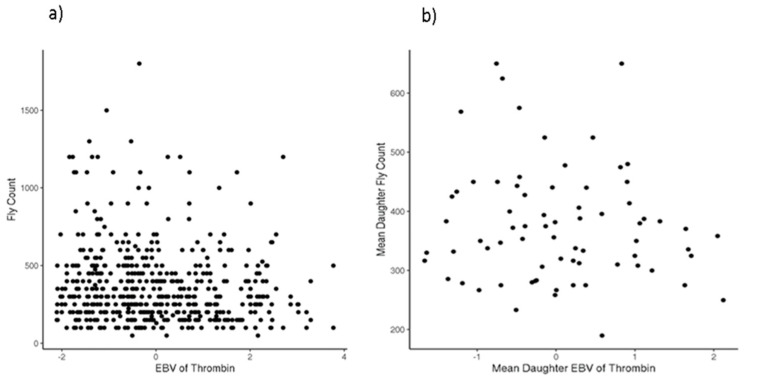
Relationship between fly count and estimated breeding values (EBV) for (**a**) individual animals and (**b**) average of sires’ daughters.

Table 3 presents the estimates of the genetic parameters of thrombin using linear and threshold models. The posterior mean of the additive variance using both models (7.12 and 1.11) indicates the presence of sufficient genetic variation in the trait. Estimates of the heritability were 0.38 and 0.39 using the linear and threshold models, respectively. The estimates of repeatability were 0.56 and 0.57 using linear and threshold models, respectively (Table 3). There is a negative correlation (−0.18) between fly count and breeding values for thrombin estimated using the linear model. These results are in concordance with the negative phenotypic association (correlation of −0.13) between thrombin and fly count (Figure 5).

## 4. Discussion

Currently, there are no reliable estimates for the onset of economic injury threshold due to horn fly abundance. Although an economic injury threshold of 200 flies per side of an animal is often reported in the literature [24], this number was not scientifically derived. Based on our data, it seems that this threshold is inaccurate, at least for the cattle used in this study. In fact, the vast majority of the animals have much higher fly abundance (Table 2). This could be the result of a correlative response due to selection for growth rate. Animals with low resistance to and tolerance of horn fly see their growth rate more pronouncedly affected, limiting their probability of being selected. Another potential explanation is the more frequent use of fly control tools including life cycle interruption through manure management, inclusion of growth regulators in the host diet, and reduction in the adult fly population through the use of insecticides currently compared to 30 or 40 years ago.

Although the concordance between evaluators was reasonable, it could be improved if the different measurements were taken at the same time. However, that is not always possible under pasture conditions. Given the complexity of the task, it is expected that a non-negligible variation in fly count assessment between evaluators would be observed. The average count across several evaluators tends to be less noisy when animals were placed in abundance classes. Unfortunately, that is not a practical solution. However, evaluators had much higher concordance (86%) in classifying animals within three fly abundance classes (High > 75 percentile; 25 < Moderate < 75 percentile; and Low < 25 percentile). Using discrete classes can increase consistency across evaluators with implications on the biological definition of the trait and the statistical methodology needed for its analysis.

As seen in Figure 1, the marked phenotypic variation in fly abundance between sire families under similar environmental and management conditions points towards the possibility for direct or indirect genetic reasons for HF attraction in cattle. Although the genetic basis of the trait remains largely unknown, the phenotypic variability observed in this study seems to support previous results. Heritability estimates based on small scale studies ranged between 10 and 80% [16,17,18]. Similar results, although with lower heritabilities, were observed for self-reported wheal size, itch intensity, and attractiveness to mosquitoes in humans [19].

The majority of the assessed animals had a horn fly count below 500 with substantial variation in predicted thrombin. However, animals with high fly abundance (>800 flies) tend to have significantly lower predicted thrombin, as indicated in Figure 3. This pattern seems to indicate that animals that attract a large number of flies consistently have a low blood thrombin. However, the opposite is not necessarily true. This behavior seems to suggest the potential existence of a threshold for blood thrombin concentration below which fly abundance increases substantially. This is not surprising as thrombin plays an important role in the activation of several pro-coagulation factors through the conversion of fibrinogen to fibrin. The linear relationship between predicted thrombin and horn fly count measured by the Pearson correlation indicates a relatively weak association. Based on the results presented in Figure 3, it is likely that the relationship between thrombin and horn fly abundance is nonlinear. This is partially supported by the clear difference in fly counts between the low and high thrombin classes (Figure 4).

Estimates of heritability clearly indicate the potential for selection on thrombin concentration in the blood to indirectly reduce the abundance of horn flies. To the best of our knowledge, this is the first study to report estimates of the heritability of thrombin in livestock. However, there are many studies on the genetic determinants of thrombin and related phenotypes in humans due to their relationships with cardiovascular diseases, venous thrombosis, and gait [25]. Prothrombin (a precursor of thrombin) showed a high heritability ranging from 0.49 to 0.70 [26,27]. Similarly, several thrombin-related traits, including thrombin peak, lag time, and venous thromboembolism, were highly heritable, with heritability greater than 0.5 [28]. Even after consideration of the relatively large posterior standard deviations, our estimates of the heritability of thrombin seem to be on the lower end compared to estimates in humans. Our estimates of repeatability were, as expected, higher than the portion of the variance explained by the household common environmental factors used in human studies. Though standard errors of the estimates for the genetic and permanent environment variance are large for both methods due to the small sample size, the estimates of the variance components are reasonable. Standard deviations associated with estimates of the heritability of thrombin in our study (Table 3) were similar or even smaller than those reported for prothrombin in several human studies [28].

There is a noticeably lower density of high fly counts among those animals with high estimated breeding values for thrombin (Figure 5a). This suggests that high levels of thrombin seem to deter high fly load; yet, the inverse trend that low levels of thrombin imply high fly count does not necessarily appear to be true and there are very likely additional biological factors (e.g., hair follicle density, skin thickness, other blood clotting factors, or behavioral avoidance) deterring flies in such cases where both thrombin and fly count are low. This trend between fly count and thrombin-estimated breeding values appears consistent within sire families as well (Figure 5b), though the small number of sire families in these data must be kept in mind when interpreting this trend.

It is worth mentioning that the impact of horn flies on economically important traits is observed when the fly abundance exceeds a certain threshold (e.g., 200 flies on one side of the animal). Thus, when using thrombin as a proxy for direct horn fly counts, treating it as a categorical trait could be an effective approach. Collectively, these results seem to indicate that thrombin could be used as a proxy for genetic selection to reduce HF infestations and that it has a reasonable genetic basis which could be harnessed for improvement in the response to HF.

## 5. Conclusions

The lack of a reliable automated system to evaluate horn fly abundance under field conditions and the logistical difficulties and uncertainty associated with the subjective assessment of fly count using trained evaluators drive the need to develop alternative practical methods to assess HF abundance on beef cattle. Thrombin, a blood enzyme involved in the coagulation pathway, seems to have a small negative correlation with HF abundance. Furthermore, high levels of thrombin may deter flies and be indicative of low fly counts. However, low levels of thrombin seem to be poorer indicators of horn fly count. Thrombin is moderately heritable and can serve as a proxy in selecting against horn fly attraction. However, a more efficient selection program might require the consideration of additional biological factors with a potential association with horn fly abundance. In fact, we are currently assessing digitally collected behavior parameters on the prediction of fly abundance. We believe that thrombin could be used in combination with other biological factors, not only to estimate fly abundance but also as a proxy for the genetic selection of lower fly abundance on beef cattle.

## Figures and Tables

**Table 1:** Estimates of the regression coefficients for thrombin prediction in the standards as a function of optical density (OD) reads in each of the three days.

Day	β_0_	β_1_	β_2_
1	1443.2	1439.7	364.3
2	1511.9	2160.4	724.4
3	1794.7	2107.9	626.4

**Table 2:** Summary description of subjective assessment of horn fly abundance on both farms for both evaluation dates.

Farm	Number of Animals	Mean	Min	Max	SD
Eatonton Beef Research Unit Northwest Georgia REC	210 145	489 384	50 100	1500 750	296 162

**Table 3:** Genetic parameters of thrombin using linear and threshold models.

Model	Variances			Heritability	Repeatability
	Additive	Permanent	Residual		
**Linear**	7.12 (1.92)	3.36 (1.56)	8.15 (2.85)	0.38 (0.09)	0.56 (0.05)
**Threshold**	1.11 (0.39)	0.52 (0.33)	1.20 (0.21)	0.39 (0.09)	0.57 (0.06)

## Data Availability

These data were obtained during the study and are not available to the public at this time.
